# Rapid Nucleic Acid Detection of *Listeria monocytogenes* Based on RAA-CRISPR Cas12a System

**DOI:** 10.3390/ijms25063477

**Published:** 2024-03-20

**Authors:** Yujuan Yang, Xiangxiang Kong, Jielin Yang, Junxin Xue, Bing Niu, Qin Chen

**Affiliations:** 1 School of Life Sciences, Shanghai University, Shanghai 200444, China; yangyj@shu.edu.cn; 2 Institute of Translational Medicine, Shanghai University, Shanghai 200444, China; kongxiangxiang@shu.edu.cn; 3 School of Medicine, Shanghai University, Shanghai 200444, China; 4 Technical Centre for Animal, Plant and Food Inspection and Quarantine of Shanghai Customs, Shanghai 200135, China; yangjielin@customs.gov.cn (J.Y.); xuejunxin@126.com (J.X.)

**Keywords:** RAA-CRISPR/Cas12a, nucleic acid detection, *Listeria* *monocytogenes*, fluorescence system

## Abstract

*Listeria monocytogenes* (*L. monocytogenes*) is a food-borne pathogenic bacteria that frequently contaminates animal-derived food and low-temperature preserved food. *Listeriosis* caused by its infection has a high mortality rate and poses a serious threat to human health. Therefore, it is crucial to establish a sensitive, rapid and easy-to-operate technique. In this study, a Recombinase Aided Amplification (RAA) assisted CRISPR/Cas12a (RAA-CRISPR/Cas12a) fluorescence platform was established for highly sensitive nucleic acid detection of *L. monocytogenes*. The established RAA-CRISPR/Cas12a showed high sensitivity and high specificity, with the sensitivity of 350 CFU/mL and 5.4 × 10^−3^ ng/μL for pure bacterial solution and genomic DNA, and good specificity for 5 strains of *Listeria* spp. and 14 strains of other common pathogenic bacteria. *L. monocytogenes* could be detected at an initial concentration of 2.3 CFU/25g within 2 h of enriching the beef in the food matrix, and this method could be applied to food samples that were easily contaminated with *L. monocytogenes* The results of RAA-CRISPR/Cas12a could be observed in 5 min, while the amplification was completed in 20–30 min. The speed and sensitivity of RAA-CRISPR/Cas12a were significantly higher than that of the national standard method. In conclusion, the RAA-CRISPR/Cas12a system established in this study has new application potential in the diagnosis of food-borne pathogens.

## 1. Introduction

Nowadays, people’s demand for food safety and food quality is gradually increasing as food safety problems are also increasing year by year, especially the frequent occurrence of food-borne pathogenic microorganisms, which poses a serious threat to the development of the food industry [1,2,3]. *Listeria monocytogenes* (*L. monocytogenes*) is one of the foodborne pathogenic bacteria that is widely found in nature. *L. monocytogenes* causes zoonotic diseases, and human infections can lead to sepsis, meningitis, etc., with a mortality rate of 27% to 44% after an outbreak [4,5]. At present, the detection and identification of *L. monocytogenes* in food is mostly limited to traditional isolation and culture methods [6]. These methods have many disadvantages, such as low detection sensitivity and tendency to miss target and miscellaneous bacteria. In addition, bacteria counts are varied due to different operator and counting methods, and the methods time-consuming and cumbersome, making it unsuitable for real-time monitoring. In recent years, the Polymerase Chain Reaction (PCR) detection approach based on molecular biology and Enzyme-linked Immunosorbent Assay (ELISA) detection based on immunology have been widely studied. Real-time quantitative PCR (qPCR) is a nucleic acid detection method widely used in laboratories at present, with high sensitivity, strong specificity and quantitative detection [7,8]. However, the instrument is expensive and requires specialist personnel to operate [9]. The thermostatic nucleic acid detection technology represented by loop-mediated isothermal amplification (LAMP) [10], Surface enhanced Raman scattering (SERS) detection [11] and biosensor detection [12], etc., has a complex operation process, high technical requirements and needs expensive equipment, and these detection methods are difficult to be realized in common detection institutions or experimental platforms. Therefore, it is necessary to design and develop a detection method for *L. monocytogenes* with high specificity, high sensitivity and easy operation.

**Figure 1:**
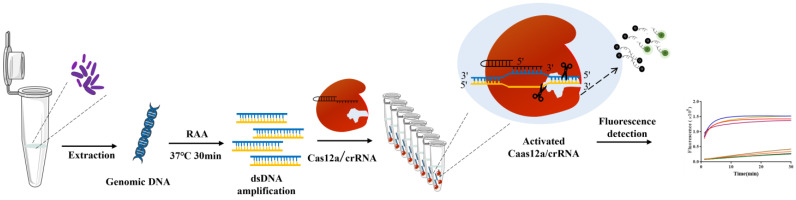
Schematic diagram of fluorescence platform based on RAA CRISPR/Cas12a for *L. monocytogenes* detection.

In recent years, the clustered regularly interspaced short palindromic repeats/associated protein (CRISPR/Cas) system has been widely used for genome editing or specific and ultra-sensitive detection of bio-macromolecules due to its ability to shear foreign genomes [13]. The CRISPR/Cas system is a unique adaptive immune system in prokaryotic cells, which works based on the nucleic acid recognition function guided by simple CRISPR RNA (crRNA) [14]. The shearing activity of the Cas protein is triggered when the crRNA binds to a complementary target DNA or RNA fragment. Cas nucleases such as Cas9, Cas12 and Cas13 have been reported to be used for the nucleic acid detection of *Salmonella, Staphylococcus aureus*, *Escherichia coli O157:H7* and other important pathogens [15,16,17]. In addition to bacteria, nucleic acids have been reported using CRISPR/Cas system to detect viruses such as human papilloma virus (HPV), hepatitis B virus (HBV) and Severe Acute Respiratory Syndrome Coronavirus 2 (SARS-CoV-2) [18,19,20]. Among these nucleases, Cas12a can directly cleave single-stranded DNA (ssDNA) of any sequence without DNA transcription. Combined with RAA, LAMP and other nucleic acid amplification technologies, the CRISPR/Cas12a system can be established for nucleic acid detection [21,22]. RAA is a new isothermal nucleic acid amplification method. It has also been used for detection in many previous studies, requiring recombinant enzyme uvsX, single-strand DNA binding protein (SSB) and DNA polymerase to achieve nucleic acid detection of the target pathogen [23]. Therefore, this study applied the CRISPR/Cas12a system (RAA-CRISPR/Cas12a) combined with RAA technology to detect *L. monocytogenes*, providing a theoretical basis for effectively control and prevention of the spread and development of *L. monocytogenes*. The detection process diagram is shown in Figure 1.

## 2. Results

### 2.1. Optimization of RAA-CRISPR/Cas12a Detection System

**Figure 2:**
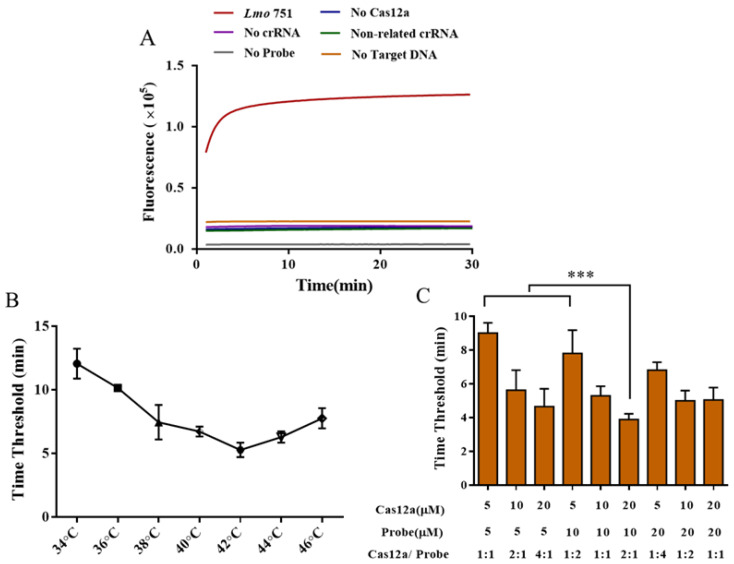
Optimization of RAA-CRISPR/Cas12a detection system. (**A**) Feasibility evaluation of RAA-CRISPR/Cas12a system; (**B**) screening of reaction temperature for RAA-CRISPR/Cas12a system; (**C**) effect of different proportions of Cas12a and ssDNA-FQ on the reaction efficiency in the system. *** represents *p* < 0.01.

The CRISPR/Cas12a system was used to detect the *hly* (NC_003210.1) gene of *L. monocytogenes* amplified by RAA, and the feasibility test of RAA primers and CRISPR/Cas12a system components was conducted (see Figure 2). As can be seen from Figure 2A, ssDNA-FQ reporter fluoresces and is detected by the system when the correct target DNA and Cas12a/crRNA complex are present in the assay system, demonstrating that the RAA-CRISPR/Cas12A system constructed in this study can be used to detect *L. monocytogenes*. When the reaction system was screened for detection temperature (Figure 2B), the minimum time threshold for amplification was 5.3 when the amplification temperature was 42 °C, indicating a rapid amplification efficiency at this temperature. The different ratios of Cas12a and ssDNA-FQ influenced detection efficiency (Figure 2C). When the volume of Cas12a and probe were 20 μM and 10 μM, respectively, i.e., 2:1, the minimum amplification time threshold was 3.89, which was the best amplification efficiency under this condition. Therefore, the subsequent experiment was conducted using the reaction temperature of 42 °C and the reaction system of ssDNA-FQ (10 μM) and Cas12a (20 μM).

### 2.2. Evaluation of the Sensitivity of the L. monocytogenes RAA-CRISPR/Cas12a Detection System

**Figure 3:**
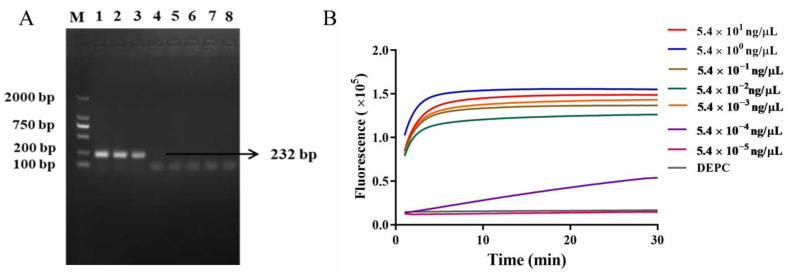
Sensitivity analysis of RAA-CRISPR/Cas12a detection system (**A**) Agarose gel electric RAA amplification map (M: 2000 bp DNA Marker; Lane 1–7: 5.4 × 10^7^ CFU/mL to 5.4 × 10^1^ CFU/mL *L. monocytogenes* solution; Lane 8: Nuclease free water); (**B**) DNA amplification of RAA-CRISPR/Cas12a bacterial genome.

The results of the RAA amplification assay for *L. monocytogenes* (ATCC BAA 751) from 5.4 × 10^1^ ng/μL to 5.4 × 10^−5^ ng/μL are shown in Figure 3. The susceptibility of *L. monocytogenes* was detected by RAA amplification, and the results showed that the sensitivity of agarose gel electrophoresis after RAA amplification was 10^5^ CFU/mL (Figure 3A). The detection sensitivity of simple RAA was low, and the products amplified by RAA were combined with CRISPR/Cas12a technology to construct a RAA-CRISPR/Cas12a detection system (Figure 3B). Figure 3B showed that the genome DNA of *L. monocytogenes* from 5.4 × 10^1^~5.4 × 10^−3^ ng/μL showed S-type amplification curve, while the nucleic acid of *L. monocytogenes* at 5.4 × 10^−4^ ng/μL showed no amplification curve, indicating that the detection limit of *L. monocytogenes* genomic DNA by the RAA-CRISPR/Cas12a detection system reached 5.4 × 10^−3^ ng/μL, equivalent to a bacterial concentration of 350 CFU/mL.

### 2.3. Evaluation of the Specificity of Intergeneric and Interspecific RAA-CRISPR/Cas12a Detection System for L. monocytogenes

**Figure 4:**
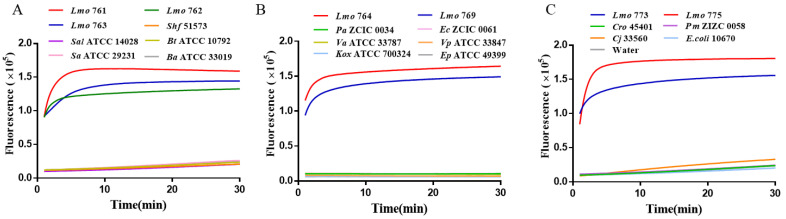
Detection of intergeneric RAA-CRISPR/Cas12a of *L. monocytogenes*. (**A**) Lmo 761, 762, and 763 were co-tested with other bacteria. (**B**) Lmo 764, and 769 were co-tested with other bacteria. (**C**) Lmo 773 and 775 were co-tested with other bacteria.

An RAA-based CRISPR/Cas12a method was used to detect the specificity of *L. monocytogenes*. A total of 32 bacterial strains including 13 *L. monocytogenes*, 5 *Listeria* spp. strains and 14 other common pathogenic bacteria were selected (Table 1). These strains were selected as controls to verify the specificity of this method. Figure 4 shows the detection of RAA-CRISPR/Cas12a and 14 common pathogenic bacteria including *L. monocytogenes*, *Shigella*, *Staphylococcus aureus*, *Bacillus cereus*, etc. Only *L. monocytogenes* showed an S-type amplification curve with a significantly enhanced fluorescence signal, while the amplification results of the other 14 common pathogenic bacteria did not differ from the negative control, and they were all negative.

**Figure 5:**
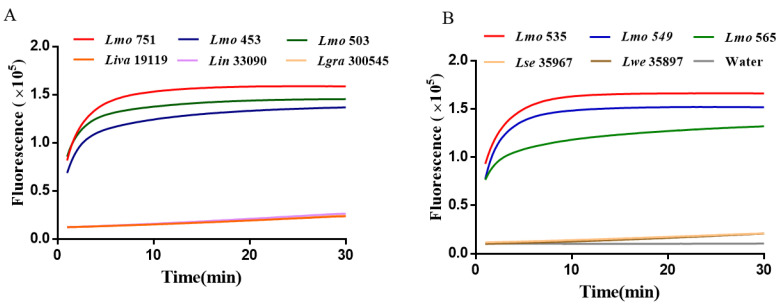
Detection of interspecific RAA-CRISPR/Cas12a specificity of *L. monocytogenes*. (**A**) Lmo 751, 453, and 503 were co-tested with other bacteria. (**B**) Lmo 535, 549, and 565 were co-tested with other bacteria.

These results indicated that the RAA-CRISPR/Cas12a assay system is independent and limited by other pathogenic bacteria, and had good specificity. The specificity of the method was further evaluated by elevating the genus-to-genus RAA-CRISPR/Cas12a detection system to the species level for the detection of other species of the genus *Listeria* (Figure 5). The results showed that only 6 *L. monocytogenes* showed positive amplification, while *Listeria ivanovii*, *Listeria innocua*, *Listeria grayi*, *Listeria seeligeri*, *Listeria welshimeri* and the negative control without nuclease showed a stable and low fluorescence signal. These results indicated the role of the RAA-based CRISPR/Cas12a assay in distinguishing *L. monocytogenes* from non-target species, and the RAA-CRISPR/Cas12a assay had a good specificity for the detection of *L. monocytogenes*.

### 2.4. Detection of L. monocytogenes in Beef Substrate and Comparison with National Standard Method

**Figure 6:**
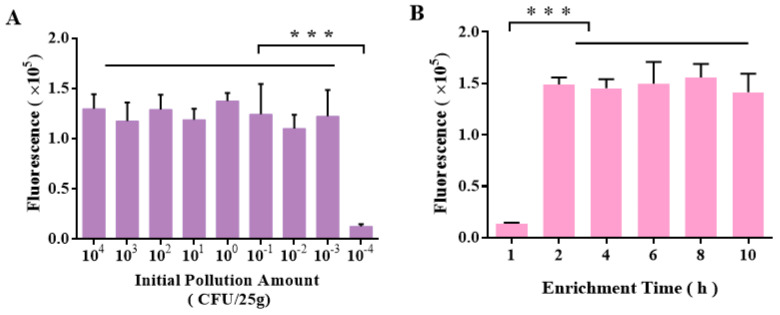
Detection results of *L. monocytogenes* with different initial contamination levels (**A**) and different enrichment times (**B**). *** represents *p* < 0.01.

Artificially contaminated beef samples containing *L. monocytogenes* were tested for the detection limit and rapid detection performance of this method for artificial inoculation and compared with the national standard method (Table 2). The results are shown in Figure 6. The detection limit of the traditional national standard method in the beef matrix was 2.3 × 10^−2^ CFU/25 g, while the RAA-CRISPR/Cas12a method could detect *L. monocytogenes* with the artificial inoculation amount of 2.3 × 10^−3^ CFU/25 g, which was lower than the detection limit of the national standard method. The beef samples with the initial contamination level of 2.3 × 10^1^ CFU/25 g were used to compare the incubation time (Figure 6A). The target strain could only be detected after 4 h of incubation with a conventional culture method, whereas the *L. monocytogenes* could be detected after 2 h of incubation with the RAA-CRISPR/Cas12a method. These results indicate that the RAA-CRISPR/Cas12a assay platform established in this study can rapidly detect beef products contaminated with *L. monocytogenes*, with a lower detection limit (2.3 × 10^−3^ CFU/25 g) than the conventional culture method, and a much shorter culture time, avoiding the disadvantage of the conventional culture method, which usually takes long time.

### 2.5. Detection of L. monocytogenes in Actual Samples with RAA-CRISPR/Cas12a

Finally, a total of 28 real samples were selected to validate our proposed detection approach (Table 2). All selected samples were tested with the RAA-CRISPR/Cas12a system, with two positive samples detected in cattle plate tendon and skimmed milk powder 2, and the remaining samples were negative. The traditional national standard method was also used for the detection of 28 samples, and the results were consistent with the results of RAA-CRISPR/Cas12a. It is suggested that the established RAA-CRISPR/Cas12a *L. monocytogenes* platform could be applied to the detection of actual samples, and provided a new method for the rapid detection of the strain.

## 3. Discussion

In recent years, CRISPR/Cas system detection technology has attracted widespread attention in the field of real-time detection of pathogenic bacteria nucleic acids due to its accurate targeting, high specificity and multiple testing [15,24,25]. The Cas12a protein-targeted and trans-cleaved single-stranded DNA system is simple and efficient. Based on this feature, pathogen nucleic acid detection has been successfully achieved, significantly improving the sensitivity of instant food detection products [26]. *L. monocytogenes* is one of the most dangerous foodborne pathogenic microorganisms that widely exists in nature [27]. The pathogen is highly tolerant of various extreme environments and is adept at spreading through various channels. It can directly cause contamination of milk, dairy products, meat and its products, seafood and aquatic products, posing challenges to food safety regulation [28]. Therefore, an accurate, rapid, and economical real-time detection technology is of great value for the food safety and quarantine inspection of the strain. In this study, we developed a Cas12a-mediated DNA detection technology that integrates RAA into the CRISPR/Cas12a system to detect *L. monocytogenes* specifically and efficiently in food, with faster detection and lower detection limit than the Chinese national standard method (GB/T 4789.30-2016). This method has several characteristics. Firstly, the reaction volume of CRISPR/Cas system is 20 μL, which allows for adequate amplification efficiency of nucleic acid and reduced reaction cost. Secondly, this system is a rapid and sensitive on-site detection technology that is simple to operate. It allows the addition of 1 μL of RAA product to the CRISPR/Cas12a system at room temperature to measure fluorescence intensity in approximately 10 min. It can be used to rapidly detect the low-level nucleic acid content in foods on site. Thirdly, specific sequence recognition, crRNA of about two dozen short nucleotides is designed, which combines with the RAA-specific amplification to make it highly specific. Fourth, the method is suitable for rapid detection of foodborne pathogens in the field or in poorly equipped laboratories in a portable thermostatic device. 

It can be seen from Figure 3 that this method has a high detection sensitivity (350 CFU/mL) and a lower detection limit than the RAA gel method and the national standard method. Previously, a nucleic acid amplification technology using the CRISPR/Cas method for the detection of *L. monocytogenes* has been developed. Lingyi Wu et al. [29] can detect 38 CFU/mL of *L. monocytogenes* genomic DNA using the CRISPR/Cas9 method combined with electrochemiluminescence with high sensitivity, but the incubation time of this process alone is as long as 1 h. In addition, Cas12a selected in this study has both cis- and trans-cleavage activities compared to the cis-cleavage activity of Cas9 protein, providing higher turnover efficiency and enabling specific detection of target sequences [30]. Moreover, this CRISPR/Cas12a method has the advantage of being instrument-independent. The operation of CRISPR/Cas12a only needs a simple thermostatic operation, and even a portable fluorescence analyzer can perform the rapid detection on site. Compared with the traditional method, it is more suitable for the detection of actual samples. Shi et al. [22] established a LAMP-based method for the thermostatic detection of *Shigella* using CRISPR/Cas12a. Compared with the method established here, the reaction temperature of the method required 60 ℃, and LAMP required the design of multiple primers. However, RAA-CRISPR/Cas12a did not only have a low reaction temperature, but also required only one pair of primers to specifically complete the detection. At the bottom of the food testing industry chain, laboratories with poor medical facilities and poor conditions can be promoted where urgent diagnosis is needed.

Our CRISPR/Cas12a approach demonstrates that CRISPR/Cas12a has great potential for developing the next-generation nucleic acid detection biosensors. In CRISPR/Cas12a system established in this study, in screening the optimal reaction temperature for Cas12a, it was found that amplification efficiency was faster at 42 °C rather than the traditional 37 °C [26], probably due to the high activity of the Cas12a enzyme at this temperature. The reaction temperature of RAA amplification method was 39 °C, which was like the reaction temperature of CRISPR/Cas12a method established in this study. The aforementioned methods can be linked together to complete nucleic acid amplification and fluorescence detection in a single reaction tube, avoiding the complexity of intermediate operations and waste of reagents [31,32]. Furthermore, this method can be combined with the transverse flow test strip technology so that the detection result can be observed by the naked eye, and the purpose of visual detection is achieved.

## 4. Materials and Methods

### 4.1. Bacterial Culture and Extraction of Genomic DNA

Thirty-four strains of bacteria were used in the experiment (Table 1) including 13 strains of *L. monocytogenes*, 5 strains of other *Listeria* spp. and 14 strains of non-*L. monocytogenes*. All strains were inoculated in brain heart infusion (BHI, Hopebio, Qingdao, China) broth (Land Bridge Technology Co., Ltd., Beijing, China), except *Vibrio phytolygium* and *Vibrio parahemolyticus* in 3% sodium chloride alkaline peptone water (Land Bridge Technology Co., Ltd., Beijing, China) and *Campylobacter jejuni* in Bolton broth (Land Bridge Technology Co., Ltd., Beijing, China). Except for *Campylobacter jejuni* cultured at 42 °C for 24 h, all strains were cultured overnight in a shaking table at 37 °C. An appropriate amount of overnight culture was taken from each bacterial strain, and the DNA was separately extracted with Tiangen bacterial genomic DNA extraction kit (Tiangen Biotech Co., Ltd., Beijing, China). The concentration and purity of genomic DNA were measured by NanoDrop 2000 spectrophotometer (Thermo Scientific, Shanghai, China), and the extracted DNA was stored in a −20 °C refrigerator for subsequent use.

### 4.2. Primer Design and crRNA Synthesis

The RAA primers, crDNA and ssDNA-FQ reporter genes were all synthesized by Sangon Biotech Co., Ltd. (Shanghai, China). The *hly* gene is a conserved virulence gene in *L. monocytogenes*, so the *hly* gene was selected as the target [33], the primers for the specific amplification of RAA were designed by DNAMAN software (DNAMAN Version 9). The fragment length and primer sequence are shown in Table 3. The upstream and downstream sequences of crDNA (Table 3) were mixed in equal proportions, denatured at 95 °C for 5 min, and incubated at 25 °C for 5 min. The double-stranded crDNA was transcribed into crRNA in vitro by HiScribe™ T7 Quick High Yield RNA Synthesis Kit (New England Biolabs), and the transcription system was as follows: nuclease free water 12 μL, 10× Reaction Buffer 2 μL, dNTP Mix 2 μL (10 Mm), crDNA 2 μL, T7 RNA polymerase and total Reaction system 20 μL. The above systemsz were mixed and incubated at 37 °C for 3 h. After transcription, 1U DNase I (Vazyme, Nanjing, China) was added and incubated at 37 °C for 15 min to remove trace residual DNA. High-purity crRNA was obtained after purification with Tianmo RNA Purification kit (Beijing, China) and stored at −80 °C for reserve.

### 4.3. RAA Amplification and CRISPR/Cas12a Detection

The RAA was detected by an RAA nucleic acid amplification kit (Jiangsu Qitian Gene Biotechnology Co., Ltd., Wuxi, China). Each reaction consisted of 25 μL RAA buffer V, 16.5 μL nuclease-free water and 2.5 μL magnesium acetate, 2 μL primer (10 μM), 2 μL DNA template. The reaction tube was heated at 39 °C for 20 min for RAA reaction. Subsequently, 50 μL phenol/chloroform (1:1) was added into the test tube, mixed well, centrifuged at 12,000 rpm for 1 min and the purified RAA product was detected by 2.0% agarose gel electrophoresis. The purified RAA product was developed by an automatic gel imaging system (Thermo Fisher Scientific Co., Ltd., Shanghai, China). The RAA-CRISPR Cas12a System mixture (20 μL) contained 2 μL Cas12a-ssDNA Buffer (10×) (Novoprotein Scientific Co., Ltd., Shanghai, China), 2 μL RAA products, 1 μM crRNA, 20 μM LbaCas12a (Novoprotein Scientific Co., Ltd., Shanghai, China), 10 μM ssDNA-FQ reporter and nuclease-free water were supplemented 20 μL, with nuclease-free water replacing the RAA product for the blank control. In blank control, nuclease free water was used to replace RAA products, and the system was slightly adjusted during condition screening. The fluorescence signal was monitored on an isothermal amplification platform (GenieII machine) (Beijing Suntrap Science & Technology Co., Ltd., Beijing, China) and the fluorescence intensity was measured every 15 s.

### 4.4. Evaluation of Specificity and Sensitivity of RAA-CRISPR/Cas12 System

To evaluate the specificity of this method, an interspecific and intergeneric detection of *L. monocytogenes* were performed. There were 13 *L. monocytogenes* strains, 5 other *Listeria spp.* strains and 14 non-*Listeria* strains (Table 1). First, 7 *L. monocytogenes* and 14 non-*L. monocytogenes* were tested for inter-genus specificity, and the remaining 6 *L. monocytogenes* were evaluated for inter-species specificity with 5 other *Listeria* spp. strains. The RAA amplification product DNA of the above 32 strains was simply added to the CRISPR/Cas12a system for immediate detection of fluorescence intensity. The sensitivity of the method was evaluated by gradient dilution with multiple dilution of *L. monocytogenes* (ATCC BAA 751) bacterial solution from 10^7^ to 10^1^ CFU/mL and genomic DNA from 5.4 × 10^1^ ng/μL to 5.4 × 10^−5^ ng/μL. The sensitivity of the method was evaluated by gradient dilution by diluting the bacterial solution of *L. monocytogenes* (ATCC BAA 751) to obtain genomic DNA with DNA concentrations ranging from 5.4 × 10^1^ ng/μL to 5.4 × 10^−5^ ng/μL.

### 4.5. Validation of RAA-CRISPR/Cas12a in Beef Substrates and Detection in Real Products

Beef was selected for matrix validation. Fresh beef samples were purchased from local supermarkets and tested by RAA-CRISPR/Cas12a. Ten 25 g beef samples (UV irradiation for 45 min to ensure no bacterial contamination) were put into sterile bags, each with 225 mL of LB_1_ enrichment solution, crushed and mixed in homogenizer. Among them, nine samples were added with 2.3 × 10^4^~10^−4^ CFU/25 g *L. monocytogenes*, and one sample without *L. monocytogenes* was used as negative control. After incubation at 30 °C for 24 h, 0.1 mL sample bacterial solution was added to 10 mL LB_2_ enrichment broth for incubation at 30 °C for 18 h. The enrichment solution was centrifuged at 12,000 rpm for 2 min to extract genomic DNA. 2.3 × 10^1^ CFU/25 g *L. monocytogenes* was selected, and its enrichment solution was centrifuged (12,000 rpm, 2 min) at the 1st, 2nd, 4th, 6th, 8th and 10th hours to extract genomic DNA, and then the time of incubation was screened. At the same time, the Chinese national standard method (GB/T 4789.30-2016) was used for comparative detection of *L. monocytogenes*.

In the actual sample testing, a total of 28 copies of goods were selected, in addition to tap water were purchased in the local supermarket. Then, 25 g of meat and seafood products were sampled, and 100 mL of juice and yoghourt were sampled. After homogenization, 225 mL LB_1_ bacterial enrichment solution was added to culture at 30 °C for 24 h, and 0.1 mL sample solution was added to 10 mL LB_2_ bacterial enrichment solution for 30 °C culture for 18 h. The enriched solution was centrifuged (12,000 rpm, 2 min) to extract genomic DNA for RAA-CRISPR/Cas12a analysis. The Chinese national standard method (GB/T 4789.30-2016) was also used to test 28 actual samples.

## 5. Conclusions

In conclusion, we developed the RAA-CRISPR/Cas12a fluorescent platform for rapid, specific and sensitive nucleic acid detection of *L. monocytogenes* in this study. By integrating RAA into the CRISPR/Cas12a system, the proposed method showed good specificity for the detection of target bacteria, which could be completed within 30 min. The detection limits of bacterial concentration and genomic DNA were 350 CFU/mL and 7.5 × 10^−3^ ng/μL, respectively, showing higher sensitivity than those of the RAA gel electrophoresis method and national standard method. Importantly, the detection result of *L. monocytogenes* in the added food samples shows that the proposed method can quickly detect the beef samples polluted by *L. monocytogenes*, and only needs 2 h enrichment. Therefore, the RAA-CRISPR/Cas12a method in this study has a good development prospect in the field of diagnosis of *L. monocytogenes*.

## Figures and Tables

**Table 1:** Strains for *L. monocytogenes* specific analysis.

No.	Species	Strains	Detection Results
1	*L. monocytogenes*	^a^ ATCC BAA 751	+
2	*L. monocytogenes*	Lmo 453 (Laboratory isolate)	+
3	*L. monocytogenes*	Lmo 503 (Laboratory isolate)	+
4	*L. monocytogenes*	Lmo 535 (Laboratory isolate)	+
5	*L. monocytogenes*	Lmo 549 (Laboratory isolate)	+
6	*L. monocytogenes*	Lmo 565 (Laboratory isolate)	+
7	*L. monocytogenes*	Lmo 761 (Laboratory isolate)	+
8	*L. monocytogenes*	Lmo 762 (Laboratory isolate)	+
9	*L. monocytogenes*	Lmo 763 (Laboratory isolate)	+
10	*L. monocytogenes*	Lmo 764 (Laboratory isolate)	+
11	*L. monocytogenes*	Lmo 769 (Laboratory isolate)	+
12	*L. monocytogenes*	Lmo 773 (Laboratory isolate)	+
13	*L. monocytogenes*	Lmo 775 (Laboratory isolate)	+
14	*Listeria ivanovii*	ATCC 19119	−
15	*Listeria innocua*	ATCC 33090	−
16	*Listeria grayi*	ATCC 700545	−
17	*Listeria seeligeri*	ATCC 35967	−
18	*Listeria welshimeri*	ATCC 35897	−
19	*Shigella flexneri*	ATCC 51573	−
20	*Salmonella enteritidis*	ATCC 14028	−
21	*Bacillus thuringiensis*	ATCC 10792	−
22	*Staphylococcus aureus*	ATCC 29213	−
23	*Bacillus cereus*	ATCC 33019	−
24	*Pseudomonas aeruginosa*	0034 (Laboratory isolate)	−
25	*Enterobacter cloacae*	0061 (Laboratory isolate)	−
26	*Vibrio alginolyticus*	ATCC 33787	−
27	*Vibrio parahemolyticus*	ATCC 33847	−
28	*Klebsiella oxytoca*	ATCC 700324	−
29	*Cronobacter sakazakii*	ATCC 45401	−
30	*Proteus mirabilis*	0058 (Laboratory isolate)	−
31	*Campylobacter jejuni*	ATCC 33560	−
32	*Escherichia coli O157:H7*	^b^ CICC 10670	−

^a^ ATCC, American Type Culture Collection; ^b^ CICC, China Center of Industrial Culture Collection.

**Table 2:** Actual samples used for RAA-CRISPR/Cas12a detection.

Sample No.	Sample Name	Test Results by National Standard Method	Crispr/cas 12a Test Results (Fluorescence Intensity × 10^5^)
1	Drumstick	−	0.3212
2	Chicken breast	−	0.3434
3	Pork collar butt	−	0.4112
4	Pig back	−	0.3718
5	Cattle shoulder rib	−	0.2998
6	Cattle plate tendon	+	1.6975
7	Neck end	−	0.3661
8	Short loin	−	0.3843
9	*Cololabis saira*	−	0.4611
10	Mackerel	−	0.3817
11	*Oncorhynchus* sp.	−	0.4712
12	Carp	−	0.3447
13	Salmon	−	0.4216
14	Prawns	−	0.3599
15	Yoghourt 1	−	0.3989
16	Yoghourt 2	−	0.4217
17	Juice 1	−	0.3556
18	Juice 2	−	0.4152
19	Beer 1	−	0.4813
20	Beer 2	−	0.3414
21	Cheese	−	0.2899
22	Pure milk	−	0.3214
23	Soymilk	−	0.4461
24	Whole milk powder 1	−	0.3245
25	Whole milk powder 2	−	0.3715
26	Skim milk powder 1	−	0.4211
27	Skim milk powder 2	+	1.7516
28	Water	−	0.3240

**Table 3:** Primers, crRNA and probes for *L. monocytogenes* RAA-CRISPR/Cas12a detection.

Description	Sequence (5′-3′)
*L. MONOCYTOGENES*-F	GTAAGTGGGAAATCTGTCTCAGGTGATGTAGA
*L. MONOCYTOGENES*-R	AGTTCCCATTGCCTATACAACAAACTTCTTAAAAG
crDNA-F	GAAATTAATACGACTCACTATAGGG
crDNA-R	ACTTCATCTTTTGCGGAGCCACCGATCTACAACAG TAGAAATTCCCTATAGTGAGTCGTATTAATTTC
probe	FAM-CACCGACGGCGAGACCGACTTT-TAMARA

## Data Availability

Data is contained within the article.
